# Rapid transfer to specialist orthopaedic ward reduces mortality in hip fracture patients, but several factors reduce the ability to achieve the < 4-hour target

**DOI:** 10.1007/s00068-026-03097-5

**Published:** 2026-02-23

**Authors:** Marios Katsanevakis, Mohamed Farag, Alan R Norrish

**Affiliations:** 1 Trust Grade SHO, Division of Surgery, Nottingham University Hospitals NHS Trust, Nottingham, UK; 2 Queen’s Medical Centre Campus, Derby Rd, Lenton, Nottingham, NG7 2UH UK; 3 CT2 in Trauma and Orthopaedics, Division of Surgery, Nottingham University Hospitals NHS Trust, Nottingham, UK; 4 Clinical Associate Professor of Trauma and Orthopaedic Surgery, NIHR Biomedical Research Centre and Academic Unit of Injury, Recovery and Inflammation Sciences, School of Medicine, University of Nottingham, Nottingham, UK

**Keywords:** Hip fracture, Neck of femur, Admission, Mortality, AMT score

## Abstract

**Purpose:**

Hip fractures are one of the most common fracture types in adults. Rapid admission to orthopaedic wards from ED (< 4 h) and optimal timing for surgery within 36 h, is linked with good patient outcomes. This study aims to identify factors that influence achievement of timely admission of elderly hip fracture patients to specialist wards at a UK tertiary centre. Additionally, it seeks to examine the impact of timely admission on 30-day mortality.

**Methods:**

This retrospective, single-centre study of a cohort of 6,170 patients aged over 60 who were admitted to our hospital’s ED with hip fractures from 22/10/2015 to 02/06/2023. Patient characteristics tables were generated with patients being categorised into early ( < = 4 h) and delayed admissions (> 4 h), and then by 30-day mortality. Logistic regression analyses were performed to identify factors associated with delayed admission to specialist wards and to assess the independent effect of admission delay (over 4 h) on 30-day mortality.

**Results:**

Out of 5937 patients for whom admission data were available, 19% (1131/5937) were admitted within 4 h. Results showed that lower AMT score (OR 0.98, 95% CI: 0.96-1.00, *p* = 0.014), out-of-hours presentation (OR 1.27, 95% CI: 1.11–1.45, *p* < 0.001), and a higher Charlson Comorbidity Index (OR 1.05, 95% CI: 1.02–1.09, *p* = 0.003) were associated with an increased chance of admission delays exceeding 4 h. Furthermore, admission delays of more than 4 h (OR 1.44, 95% CI: 1.06–1.95, *p* = 0.02) were associated with increased 30-day mortality, controlling for the other risk factors.

**Conclusion:**

Timely admission of hip fracture patients over the age of 60 is critical to reduce 30-day mortality. Low AMT score, out-of-hours presentation, and multiple comorbidities impact the timeliness of admissions in our hospital, highlighting a need to address these factors to improve care for this population.

## Introduction

Hip fractures are among the most common orthopaedic presentations to emergency departments (EDs) worldwide, particularly affecting older adults [1]. Approximately one-third of women and 17% of men will sustain a hip fracture by the age of 80 [2]. The increasing prevalence of osteoporosis and the rising proportion of older individuals in the population contribute to the projected growth in hip fracture incidence over the coming decades [2].

Early surgical intervention, ideally within 36 h of presentation, has been shown to reduce postoperative complications such as pneumonia, venous thromboembolism, and pressure ulcers, while improving mobilisation, functional recovery, and survival [3, 4].

In the United Kingdom (UK), the National Health Service (NHS) introduced a 4-hour target for transfer or discharge from the ED in 2004 [5]. Breaching this standard has been associated with increased mortality: a retrospective study of over 14 million ED presentations reported a 35% increase in 30-day mortality among patients who exceeded the 4-hour threshold (Odds Ratio [OR] 1.35, 95% Confidence Interval [CI] 1.33–1.37, *p* < 0.001) [6]. Similarly, a study of 3,266 hip fracture admissions demonstrated that delayed transfer was independently associated with increased 60-day (Hazard Ratio [HR] 1.29, 95% CI 1.04–1.59, *p* = 0.019) and 90-day mortality (HR 1.36, 95% CI 1.12–1.63, *p* = 0.001) [7].

This study aims to identify the factors influencing timely (< 4 h) admission of hip fracture patients from the ED to the orthopaedic ward in our institution and to determine whether delayed admission is independently associated with increased 30-day mortality.

## Materials and methods

### Setting

This institutional, retrospective cohort study was conducted in a large tertiary trauma centre, one of the busiest in the United Kingdom (UK). The study was registered as an institutional audit project (24-600c).

### Data source

Data were extracted from the institutional database for all patients admitted with a hip fracture through the emergency department (ED) between 22 October 2015 and 2 June 2023. Our data is linked to the National Hip Fracture Database (NHFD), which provides matching services with the Office for National Statistics (ONS) for mortality data. In this dataset, a date of death is recorded only when a death is registered; absence of a recorded death date indicates survival within the follow-up period and is not a missing outcome.

### Inclusion criteria

All patients aged 60 years or above presenting with a radiologically confirmed hip fracture were eligible for inclusion.

### Variables

Extracted variables included: study ID, age, sex, Nottingham Hip Fracture Score (NHFS) [8], Charlson Comorbidity Index [9], Abbreviated Mental Test (AMT) score [10], date and time of ED presentation, pre-admission residence (own/sheltered home, residential care, or nursing home), pre-admission mobility (freely mobile without aids, mobile outdoors with one or two aids/frame, limited indoor mobility, or no functional mobility), American Society of Anaesthesiologists (ASA) grade [11], time from ED assessment to orthopaedic ward transfer, time to surgery, time to orthogeriatric review (all in minutes), length of hospital stay (days), time to death (days), and place of death (in-hospital or external).

For analysis, several variables were recoded:

- Time to admission was dichotomised as ≤ 4 h or > 4 h.
- Mortality was defined as death within 30 days of presentation.
- Presentation time was categorised as in-hours (08:00–17:00) or out-of-hours.
- Patients without a recorded time to death were considered alive at 30 days.

### Outcomes

The primary outcomes were (1) achievement of ≤ 4-hour transfer from ED to orthopaedic ward and (2) 30-day mortality.

### Statistical analysis

Statistical analysis was conducted using *R* version 4.3.2. Continuous variables were assessed for normality using density plots and reported as mean (standard deviation, SD) or median (interquartile range, IQR), as appropriate. Categorical variables were summarised as counts (n) and percentages (%). Group comparisons were performed using Welch’s two-sample *t*-test for normally distributed variables, the Wilcoxon rank-sum test for non-normally distributed variables, and Pearson’s Chi-squared test for categorical variables. A *p*-value < 0.05 was considered statistically significant.

Two logistic regression analyses—univariate and multivariable—were performed to identify independent predictors of (1) timely admission (≤ 4 h) and (2) 30-day mortality. The NHFS variable was excluded from multivariable models to avoid collinearity, as it incorporates several other covariates (age, sex, cognitive status, comorbidities, and residential status). The linearity of the log-odds assumption was verified for all continuous predictors. We opted to treat AMT and Charlson Comorbidity index as continuous variables in our models to preserve information, improve statistical power, and to avoid arbitrary cut-off points needed to convert these to categorical variables, given that the above assumption was satisfied. Missing covariate data were imputed using multiple imputation (five imputations). As the data is believed not to be missing completely at random, multiple imputation is considered superior to a complete-case analysis due to producing less biased results and offering higher statistical power [12, 13]. Variable selection followed a backward stepwise elimination process, beginning with covariates either associated with the outcome at *p* ≤ 0.2 in univariate analysis or considered clinically relevant. Final model selection was based on the Akaike Information Criterion (AIC) value and clinical interpretability. Complete case sensitivity analyses were also performed to confirm the robustness of the findings.

Results are presented as odds ratios (OR) with 95% confidence intervals (CI) and corresponding *p*-values.

### Reporting standard

This study was reported in accordance with the STROBE statement [14].

## Results

**Fig. 1:**
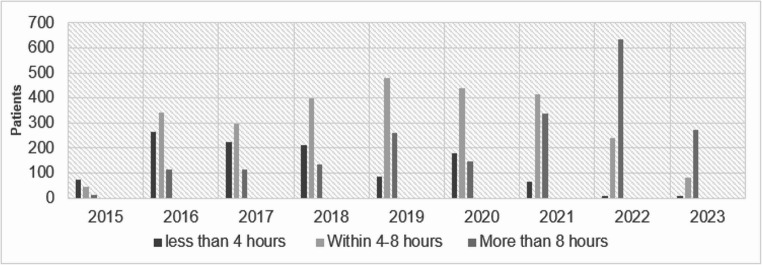
Bar plots for time from ED to ward per year

Of 6,170 patients initially included in our cohort, 5,937 (96%) had time of admission to ward data available. Additionally, 72% of the time to death data, and 10.5% of the NHFS data were missing. The remaining variables used in our analyses had missing data ranging from 0% to 4.6%. Out of the 5,937 patients with available admission time data, 1,131 (19%) achieved the 4-hour target for timely transfer to the ward. 4,137 (70%) of the patients were females. This female/male ratio remained the same within the ≤ 4 h and > 4 h groups. The mean age of our cohort was 83 years. 1,103 (19%) of patients lived in an institution (1 case unknown), and 3,175 (53%) arrived in the ED out of hours. The median Charlson comorbidity index score was 6 (IQR 4, 7), the median NHFS was 5 (4, 6), median AMT score was 8 (IQR 5), and the median time to surgery was 28 h (IQR 22). From 2015 to 2023, there was a gradual decrease in the percentage of patients who achieved ≤ 4-hour transfer to an orthopaedic ward (from 54.9% in 2015 to 1.9% in 2023) (Fig. 1).

Similar percentages could be found in the whole cohort, which also included patients with unknown time to transfer data. The above statistics are summarised in Tables 1 and 2 below, with between-group comparisons provided.

**Table 1:** Patient characteristics by achievement of ≤ 4 h transfer to the ward

Characteristic		≤ 4-hour transfer to orthopaedic ward		
	Overall, *N* = 5,937	Yes, *N* = 1,131	No, *N* = 4,806	*p*-value^*^
Sex, n (%)				0.7
Female	4,137 (70%)	794 (70%)	3,343 (70%)	
Male	1,800 (30%)	337 (30%)	1,463 (30%)	
Age, Mean (SD)	83 (9)	82 (9)	83 (9)	0.074
Pre-admission mobility, n (%)				0.023
Freely mobile without aids	2,182 (37%)	453 (40%)	1,729 (36%)	
Mobile outdoors with one aid	1,565 (27%)	306 (27%)	1,259 (26%)	
Mobile outdoors with two aids or a frame	1,658 (28%)	293 (26%)	1,365 (29%)	
Some indoor mobility, but never goes outside without help	424 (7.2%)	63 (5.6%)	361 (7.6%)	
No functional mobility	63 (1.1%)	11 (1.0%)	52 (1.1%)	
Unknown	45	5	40	
Care home resident, n (%)	1,103 (19%)	194 (17%)	909 (19%)	0.2
Unknown	1	0	1	
Charlson Comorbidity Index, Median (IQR)	6 (4, 7)	5 (4, 7)	6 (4, 7)	< 0.001
NHFS, Median (IQR)	5 (4, 6)	5 (4, 6)	5 (4, 6)	< 0.001
Unknown	571	90	481	
Year, n (%)				< 0.001
2015	133 (2.2%)	73 (6.5%)	60 (1.2%)	
2016	721 (12%)	265 (23%)	456 (9.5%)	
2017	651 (11%)	237 (21%)	414 (8.6%)	
2018	770 (13%)	210 (19%)	560 (12%)	
2019	829 (14%)	86 (7.6%)	743 (15%)	
2020	762 (13%)	178 (16%)	584 (12%)	
2021	822 (14%)	66 (5.8%)	756 (16%)	
2022	887 (15%)	9 (0.8%)	878 (18%)	
2023	362 (6.1%)	7 (0.6%)	355 (7.4%)	
Out of hours, n (%)	3,175 (53%)	548 (48%)	2,627 (55%)	< 0.001
Hours to surgery, Median (IQR)	28 (21, 43)	25 (20, 38)	29 (22, 44)	< 0.001
Unknown	141	9	132	
Hours to orthogeriatric review, Median (IQR)	22 (16, 42)	21 (15, 42)	22 (16, 41)	< 0.001
Unknown	44	8	36	
AMT score, Median (IQR)	8 (5, 10)	9 (6, 10)	8 (4, 10)	< 0.001
Unknown	80	14	66	

^*^Pearson’s Chi-squared test; Welch Two Sample t-test; Wilcoxon rank sum test (for hours to surgery, hours to orthogeriatric review, AMT score)

**Table 2:** Patient characteristics by 30-day mortality

Characteristic		30-day mortality		
	Overall, *N* = 6,170	Yes, *N* = 448	No, *N* = 5,722	*p*-value^*^
Sex, n (%)				< 0.001
Female	4,292 (70%)	251 (56%)	4,041 (71%)	
Male	1,878 (30%)	197 (44%)	1,681 (29%)	
Age, Mean (SD)	83 (9)	86 (8)	82 (9)	< 0.001
Pre-admission mobility, n (%)				< 0.001
Freely mobile without aids	2,247 (37%)	116 (27%)	2,131 (38%)	
Mobile outdoors with one aid	1,620 (26%)	104 (24%)	1,516 (27%)	
Mobile outdoors with two aids or a frame	1,730 (28%)	152 (35%)	1,578 (28%)	
Some indoor mobility, but never goes outside without help	445 (7.3%)	51 (12%)	394 (6.9%)	
No functional mobility	72 (1.2%)	11 (2.5%)	61 (1.1%)	
Unknown	56	14	42	
Care home resident, n (%)	1,134 (18%)	124 (28%)	1,010 (18%)	< 0.001
Unknown	1	0	1	
Charlson Comorbidity Index, Median (IQR)	6 (4, 7)	7 (5, 8)	5 (4,7)	< 0.001
NHFS, Median (IQR)	5 (4, 6)	6 (5, 7)	5 (4, 6)	< 0.001
Unknown	648	43	605	
Year, n (%)				0.7
2015	136 (2.2%)	11 (2.5%)	125 (2.2%)	
2016	748(12%)	52 (12%)	696 (12%)	
2017	681 (11%)	46 (10%)	635 (11%)	
2018	807 (13%)	49 (11%)	758 (13%)	
2019	856 (14%)	63 (14%)	793 (14%)	
2020	791 (13%)	60 (13%)	731 (13%)	
2021	860 (14%)	74 (17%)	786 (14%)	
2022	916 (15%)	70 (16%)	846 (15%)	
2023	375 (6.1%)	23 (5.1%)	352 (6.2%)	
Out of hours presentation, n (%)	3,288 (53%)	249 (56%)	3,039 (53%)	0.3
AMT score, Median (IQR)	8 (5, 10)	6 (1, 9)	9 (5, 10)	< 0.001
Unknown	118	13	105	
Hours to orthogeriatric review, Median (IQR)	22 (16, 42)	24 (15, 42)	22 (15, 42)	0.5
Unknown	176	38	138	
<= 4-hour transfer to ward, n (%)	1,131 (19%)	54 (13%)	1,077 (20%)	0.001
Unknown	233	33	200	
Hours to surgery, Median (IQR)	28 (21, 43)	34 (22, 48)	28 (21, 43)	< 0.001
Unknown				

^*^ Pearson’s Chi-squared test; Welch Two Sample t-test; Wilcoxon rank sum test (for hours to surgery, hours to orthogeriatric review, AMT score)

Univariate and multivariable analyses were also performed for our two outcomes (Tables 3 and 4).

**Table 3:** Logistic regression analysis of late (> 4 h) transfer from ED to ward

Characteristic	Univariate			Multivariable		
	OR^*^	95% CI^*^	*p*-value	OR^*^	95% CI^*^	*p*-value
AMT score	0.97	0.95, 0.98	< 0.001	0.98	0.96, 1.00	0.014
Out-of-hours presentation						
No	-	-		-	-	
Yes	1.28	1.13, 1.46	< 0.001	1.27	1.11, 1.45	< 0.001
Charlson Comorbidity Index	1.07	1.03, 1.10	< 0.001	1.05	1.02, 1.09	0.003
Age	1.01	1.00, 1.01	0.071			
Sex						
Female	-	-				
Male	1.03	0.90, 1.19	0.7			
Pre-admission mobility						
Freely mobile without aids	-	-				
Mobile outdoors with one aid	1.08	0.91, 1.27	0.4			
Mobile outdoors with two aids or a frame	1.22	1.03, 1.43	0.018			
Some indoor mobility, but never goes outside without help	1.50	1.13, 2.00	0.005			
No functional mobility	1.25	0.65, 2.42	0.5			
Care home resident						
No	-	-				
Yes	1.13	0.95, 1.34	0.2			
NHFS	1.09	1.04, 1.15	< 0.001			

^*^ OR: Odds Ratio. For continuous variables (AMT score, Charlson, Age, NHFS) OR represents odds of *n* + 1/odds of n; CI: Confidence Interval

**Table 4:** Logistic regression analysis of 30-day mortality

Characteristic	Univariate			Multivariable		
	OR^*^	95% CI^*^	*p*-value	OR^*^	95% CI^*^	*p*-value
Age	1.05	1.04, 1.06	< 0.001	1.03	1.01, 1.04	< 0.001
Sex						
Female	-	-		-	-	
Male	1.89	1.55, 2.29	< 0.001	2.03	1.65, 2.50	< 0.001
AMT score	0.88	0.86, 0.91	< 0.001	0.91	0.89, 0.94	< 0.001
> 4-hour transfer						
No	-	-		-	-	
Yes	1.61	1.21, 2.15	< 0.001	1.44	1.06, 1.95	0.02
Charlson Comorbidity Index	1.34	1.28, 1.40	< 0.001	1.23	1.17, 1.30	< 0.001
Pre-admission mobility						
Freely mobile without aids	-	-		-	-	
Mobile outdoors with one aid	1.26	0.96, 1.65	0.092	1.07	0.81, 1.43	0.6
Mobile outdoors with two aids or a frame	1.77	1.38, 2.27	< 0.001	1.16	0.89, 1.53	0.3
Some indoor mobility, but never goes outside without help	2.35	1.66, 3.31	< 0.001	1.51	1.04, 2.17	0.029
No functional mobility	3.35	1.71, 6.53	< 0.001	2.14	1.06, 4.33	0.033
Hours to surgery from admission	1.00	1.00, 1.00	0.7	1.00	1.00, 1.00	0.8
Hours to orthogeriatric review from admission	1.01	1.00, 1.01	< 0.001	1.01	1.00, 1.01	< 0.001
Year	1.02	0.98, 1.06	0.4	0.99	0.94, 1.04	0.7
Out-of-hours presentation						
No	-	-				
Yes	1.1	0.91, 1.34	0.3			
Care home resident						
No	-	-				
Yes	1.79	1.44, 2.22	< 0.001			
NHFS	1.59	1.48, 1.71	< 0.001			

^*^OR: Odds Ratio. For continuous variables (AMT score, Age, Charlson, Year, hours to surgery/orthogeriatric review) OR represents odds of *n* + 1/odds of n; CI: Confidence Interval

The robustness of these results has been confirmed by a complete case sensitivity analysis, which produced very similar results. Both univariate analyses reflect the findings of the t-tests and chi-square tests presented in our patient characteristics tables. Multivariable analyses were conducted, and independent associations were identified after eliminating confounding factors. Delayed (> 4-hour) transfer to the ward was strongly associated with patient AMT score (*p* = 0.014), out-of-hours presentation (*p* < 0.001), and higher Charlson comorbidity index (*p* = 0.003). More specifically, when controlling for other factors, patients have 2% lower odds (OR 0.98) of late transfer for each extra point in the AMT score, while those coming in out-of-hours had 27% higher odds (OR = 1.27). Finally, each 1-point increase in Charlson score is associated with a 5% increase in odds (OR = 1.05) for late transfer to the ward.

Patient age (*p* < 0.001), sex (*p* < 0.001), AMT score (*p* < 0.001), delayed transfer to ward (*p* = 0.02), higher Charlson score (*p* < 0.001), pre-admission mobility, and time from admission to orthogeriatric review(*p* < 0.001) were all found to be significant independent predictors of 30-day mortality in our multivariable model. Specifically, we found that 30-day mortality odds increase by 3% for every additional year in the patient’s age (OR = 1.03), 23% for every additional point in the Charlson score (OR = 1.23), and 1% for every additional hour until orthogeriatric review (OR = 1.01). Additionally, male sex was associated with 103% higher odds (OR = 2.03), AMT score with 9% lower odds (OR = 0.91) for each increasing point, and delayed admission of more than 4 h with 44% higher odds (OR = 1.44) of 30-day mortality. Regarding pre-admission mobility, only significant impairment was found to influence the 30-day mortality, when compared to full, independent mobility, with limited indoor mobility associated with 51% higher odds (OR = 1.51, *p* = 0.029), and no functional mobility with 114% higher odds (OR = 2.14, *p* = 0.033). Slightly impaired mobility was not a strong predictor of 30-day mortality compared to full, independent mobility.

While the calendar year and time from admission to surgery did not significantly affect the outcome, they have been included in the analysis, the former to account for time-period related systemic and hospital factors and the latter as a strong confounding factor.

## Discussion

This study identified several factors contributing to delayed (> 4 h) admission of elderly hip fracture patients from the emergency department (ED) to the orthopaedic ward and evaluated the impact of this delay on 30-day mortality. Among 6,170 patients, delayed transfer was associated with lower AMT score, higher Charlson comorbidity index, and out-of-hours presentation. Furthermore, delayed admission was linked to a 44% increase in 30-day mortality, even after adjusting for age, sex, AMT score, Charlson score, pre-admission mobility, time from admission to surgery, time from admission to orthogeriatric review, and calendar year. These findings are consistent with previous research. Clement et al. [7] demonstrated that delayed transfer was associated with increased mortality, although their smaller cohort (*n* = 3,266) did not reach statistical significance at the 30-day time point (HR 1.25). The larger sample size and older inclusion threshold in our study (≥ 60 years) likely enhanced statistical power. Patel et al. [15], in a national record-linkage study of 178,757 patients aged ≥ 60 years, also reported a 4% rise in 30-day mortality per 5% increase in monthly ED breaches (OR 1.04, 95% CI 1.02–1.05). Together, these studies reinforce the association between prolonged ED stay and increased short-term mortality. To our knowledge, our study is the first to identify patient-related factors that independently contribute to delayed admission among hip fracture patients.

Several explanations may underlie our findings. A low AMT score can prolong the clerking process, as obtaining an adequate history and assessment often requires collateral information from relatives or carers. Hospital policy requiring supervision for these patients may also contribute to delays before orthopaedic review. Similarly, patients with higher Charlson comorbidity index scores require more extensive medical assessment and medication reconciliation, which can lengthen their ED stay. Out-of-hours presentations may coincide with reduced staffing and limited access to imaging or specialist review, contributing to systemic delays. Although pre-admission mobility appeared significant in univariate analyses, this effect did not persist after adjustment, suggesting that other factors played a more dominant role in influencing timely admission.

Regarding our 30-day mortality analysis, several well-known factors related to survivability were found as important (age, Charlson score, AMT score, pre-admission mobility). Interestingly, time from admission to surgery was not found to be an important factor. This may reflect mediation by time to admission (i.e., pre-admission delays influencing subsequent surgical timing), reducing the apparent effect of post-admission delays. On the other hand, the time from presentation in ED to admission was found to be significant, indicating that it is an important factor contributing to overall delays to patient treatment. Time from admission to orthogeriatric input was a significant factor, indicating the importance of prompt medical optimisation. Finally, despite a deterioration in 4-hour admission performance over time, the calendar year of treatment was not independently associated with 30-day mortality. The absence of a gradual increase in 30-day mortality despite deteriorating performance against the 4-hour admission target suggests that other unmeasured hospital-level factors and concurrent changes in other aspects of hip fracture care may have mitigated the impact of delayed ward transfer.

The study’s main strengths lie in its large sample size, use of multiple imputation for missing data, and multivariable analysis, which reduced confounding and enhanced the reliability of results. It is important to mention that for 72% of our cohort, there was no “days to death” data provided. However, as our data are linked to the NHFD, which is matched with ONS, we have assumed patients with no “days to death” data to be alive. ONS is a UK government body responsible for recording all deaths in England and Wales. Even though mortality ascertainment relies on ONS linkage, the absence of a recorded date of death was assumed to indicate survival at 30 days. While this approach is consistent with national hip fracture reporting practices, it cannot fully exclude the possibility of rare linkage failures or delayed registration, which could lead to non-differential misclassification of mortality status. We, however, anticipate these occurrences to be rare and not significantly impacting our results. Multiple imputation was only applied to covariates and does not mitigate this limitation.

Nonetheless, several limitations must be acknowledged. Data on fracture type, acute medical complications, and whether the presentation was part of a major trauma pathway were unavailable. These factors are known to influence both the admission process and mortality risk and their absence introduces residual confounding, particularly in the mortality models. Additionally, our analysis for the factors that lead to delayed admissions is limited to patient-related factors; the gradual decline in our performance indicates that other, hospital-related factors should also be contributing to that. Furthermore, some findings in this study are likely to have been influenced by the COVID-19 pandemic period, which had a substantial impact on healthcare delivery, staff availability, bed capacity and admission pathways. Although calendar year was included in the multivariable analyses to account for time-period related trends, this approach cannot fully capture the heterogeneous and time-varying impact of the pandemic on admission delays or mortality outcomes. Finally, as this was a single-centre study, the findings regarding factors associated with delayed admission may not be fully generalisable to other institutions. Importantly, hospital- and system-level determinants (e.g. bed capacity, staffing levels, pathway redesigns, and seasonal pressures) were not available on our data and could not be adjusted for. These factors likely contributed both to relatively stable mortality levels over time despite worsening admission time performance, thereby limiting causal interpretation of the observed association. We also acknowledge that there is established knowledge regarding admission and transfer delays for older, frail patients, and our results are not new news; we place more emphasis on our findings related to 30-day mortality and the implications these may have for informing performance standards.

Considering these limitations, future multicentre studies with comprehensive datasets are warranted. Such studies should minimise missing mortality data uncertainty and include information on fracture type, acute medical complications, trauma status, and other hospital related factors enabling more robust survival analyses and generalisable conclusions. A multicentre approach would also help identify regional or institutional variations influencing the ability to meet the 4-hour target. Within our hospital, a detailed review of the admission pathway for hip fracture patients could further clarify modifiable sources of delay and inform quality improvement initiatives to achieve timely admission.

In conclusion, delayed admission of elderly hip fracture patients to an orthopaedic ward beyond four hours of ED presentation was independently associated with higher odds of 30-day mortality, after adjustment for measured patient-level factors, within the constraints of available data and registry-linked outcome ascertainment. This association should not necessarily be interpreted as causal due to the limitations mentioned above. Patients with lower AMT score, greater comorbidity burden, and those presenting out-of-hours are particularly vulnerable to delays. Institutional efforts should therefore focus on streamlining the admission process for these high-risk groups, while larger multicentre studies with comprehensive datasets are needed to confirm these associations and evaluate the impact of timely admission on longer-term outcomes.

## Data Availability

Data sets generated during the current study are available from the corresponding author on reasonable request.
